# Decoding options and accuracy of translation of developmentally regulated UUA codon in *Streptomyces*: bioinformatic analysis

**DOI:** 10.1186/s40064-016-2683-6

**Published:** 2016-07-04

**Authors:** Ihor Rokytskyy, Oksana Koshla, Victor Fedorenko, Bohdan Ostash

**Affiliations:** Department of Genetics and Biotechnology, Ivan Franko National University of Lviv, Hrushevskoho st. 4, Lviv, 79005 Ukraine

**Keywords:** *Streptomyces*, *bldA*, tRNA, UUA codon, Codons, Models of translation

## Abstract

**Background:**

The gene *bldA* for leucyl ${\text{tRNA}}^{\text{Leu}}{}_{\text{UAA}}$ is known for almost 30 years as a key regulator of morphogenesis and secondary metabolism in genus *Streptomyces*. Codon UUA is the rarest one in *Streptomyces* genomes and is present exclusively in genes with auxiliary functions. Delayed accumulation of translation-competent ${\text{tRNA}}^{\text{Leu}}{}_{\text{UAA}}$ is believed to confine the expression of UUA-containing transcripts to stationary phase. Implicit to the regulatory function of UUA codon is the assumption about high accuracy of its translation, e.g. the latter should not occur in the absence of cognate ${\text{tRNA}}^{\text{Leu}}{}_{\text{UAA}}$. However, a growing body of facts points to the possibility of mistranslation of UUA-containing transcripts in the *bldA*-deficient mutants. It is not known what type of near-cognate tRNA(s) may decode UUA in the absence of cognate tRNA in *Streptomyces*, and whether UUA possesses certain inherent properties (such as increased/decreased accuracy of decoding) that would favor its use for regulatory purposes.

**Findings:**

Here we took bioinformatic approach to address these questions. We catalogued the entire complement of tRNA genes from several relevant *Streptomyces* and identified genes for posttranscriptional modifications of tRNA that might be involved in UUA decoding by cognate and near-cognate tRNAs.

**Conclusions:**

Based on tRNA gene content in *Streptomyces* genomes, we propose possible scenarios of UUA codon mistranslation. UUA is not associated with an increased rate of missense errors as compared to other leucyl codons, contrasting general belief that low-abundant codons are more error-prone than the high-abundant ones.

**Electronic supplementary material:**

The online version of this article (doi:10.1186/s40064-016-2683-6) contains supplementary material, which is available to authorized users.

## Findings

The *bldA* mutants of *Streptomycescoelicolor* A3(2) were first isolated almost 40 years ago (Merrick 1976) and 11 years later were shown to carry mutations within gene for leucyl ${\text{tRNA}}^{\text{Leu}}{}_{\text{UAA}}$. (Lawlor et al. 1987) This mutation abolishes aerial mycelium formation (Bald phenotype) and antibiotic production by streptomycetes; currently *bldA* is extensively used as a tool to activate cryptic secondary metabolome (Hackl and Bechthold 2015). Codon TTA, whose decoding is controlled by *bldA*, is very rare in GC-rich *Streptomyces* genomes, and present only in genes with unknown and auxiliary functions, such as colony morphological development and antibiotic production. As accumulation of translation-competent, charged ${\text{tRNA}}^{\text{Leu}}{}_{\text{UAA}}$ is confined to late stages of growth, so does the expression of TTA-containing genes (Chater 2006). UUA codon and its cognate tRNA were long time ago suggested to form a genetic switch that operates at the level of translation (Hopwood 1987). Use of TTA for regulatory purposes is somewhat controversial. On one hand, rarity of this codon ensures that only certain genes are influenced by *bldA*-based switch. On the other hand, rare codons are thought to be associated with higher missense error rates, which would not favor their proper operation as a switch. Although it was suggested implicitly that UUA is decoded accurately, there is a number of notable exceptions. Particularly, *bldA* mutants showed no Bald phenotype on certain solid media (Hopwood 1987); several TTA-containing genes were expressed in *bldA*-deficient strains (Trepanier et al. 2002; Makitrynskyy et al. 2013), particularly when their transcription is artificially elevated (Gramajo et al. 1993). All these observations imply that efficient mistranslation of UUA codon is possible at least under some conditions. It is not known what tRNAs could potentially recognize UUA in the absence of ${\text{tRNA}}^{\text{Leu}}{}_{\text{UAA}}$ and what structural and functional peculiarities of ${\text{tRNA}}^{\text{Leu}}{}_{\text{UAA}}$ contribute to its regulatory function (Pettersson and Kirsebom 2011). Here we took bioinformatic approach to obtain new insight into this issue and to chart new directions for experimental verification.

Although there are several databases of tRNA genes, such as GtRNAdb and tRNADB-CE, they provide contradictory information on tRNA content for model strain *Streptomyces coelicolor* A3(2) and lack data on several species relevant to this work. Furthermore, available online resources do not show what kind of tRNA may decode certain codon via wobble interaction. We therefore compiled all available information on tRNA genes and their decoding capacity for six *Streptomyces* species with known cases of *bldA*-based regulation using several databases and search tools detailed in Additional file 1. It could be concluded that overall tRNA gene content is highly conserved in six analyzed *Streptomyces* genomes, although copy number of individual tRNA genes varies (Table 1). For several codons there were no acceptor tRNAs (for example, alanine codon GCT); those apparently are recognized by isoacceptor tRNAs (e.g. GCT is read by GCC isoacceptor; see Table 1), which are encoded within the analyzed genomes. It is common for all known organisms that the entire set of sense codons (61 + 1 initiator) is read by far fewer than 62 isoacceptors; extreme cases of anticodon-sparing are documented in some archaea and mycoplasmas, where only 26–33 anticodons are required to read the genetic code (Marck and Grosjean 2002). All genomes contain single tRNA gene for UUA decoding. Therefore, differences in *bldA* mutant phenotypes across different species could not be ascribed to variations in tRNA gene content. Codon UUA could be poorly recognized by phenylalanine ${\text{tRNA}}^{\text{Phe}}{}_{\text{AAA}}$ via wobble interactions (Lim and Curran 2001). However, no respective tRNA gene is present in all studied *Streptomyces* genomes. Cytidine posttranscriptionally modified with lysidine (k^2^C_34_) is known to recognize adenosine in third codon position. This kind of modification to date was described only for anticodon CAU, which normally decodes methionine codon AUG. The k^2^C_34_-containing tRNA^CAU^ is charged with isoleucine and recognizes isoleucine codon AUA. It is not possible for ${\text{tRNA}}^{\text{k2CAU}}{}_{\text{Ile}}$ to recognize codon UUA because of mismatch in second codon position. Hence, there are no tRNA genes in *Streptomyces* genomes that would allow UUA codon reading (via correct or wobble interactions) in the absence of cognate ${\text{tRNA}}^{\text{UAA}}{}_{\text{Leu}}$. We therefore looked into possibility of UUA misreading. According to Lim and Curran (2001), three anticodons could misread UUA: UAC, GAA, CAA. Of these, first two would lead to aminoacid misincorporation (Val and Phe, respectively).

**Table 1:** tRNA genes in six *Streptomyces* genomes

AA	Codon	Anticodon	Codon-anticodon recognition	tRNA genes*					
				A3(2) 63**	J1074 65	ATCC 10712 65	TK24 61	ATCC 14672 68	ATCC 27064 66
1	2	3	4	5	6	7	8	9	10
Ala (A)	GCT	*AGC*	AGC, GGC	–	–	–	–	–	–
	GCC	*GGC*	AGC, GGC	SCOt14, SCOt15	XNR_4362, XNR_4366	SVEN_t15, SVEN_t16, SVEN_t56	SLI_10014, SLI_10015	SSFG_RS23155, SSFG_RS23165	SCN_RS08355, SCN_RS08365
	GCA	*TGC*	AGC, TGC	SCOt32	XNR_3021	SVEN_t34	SLI_10033	SSFG_RS17340	SCN_RS14115
	GCG	*CGC*	TGC, CGC	SCOt26	XNR_2123	SVEN_t28	SLI_10026	SSFG_RS33025, SSFG_RS20210	SCN_RS11130
Val (V)	GTT	*AAC*	AAC, GAC	–	–	–	–	–	–
	GTC	*GAC*	AAC, GAC	*vala, valb, valg*	XNR_5303, XNR_5304, XNR_5305	SVEN_t6, SVEN_t7, SVEN_t8	SLI_10007	SSFG_RS28215, SSFG_RS28210, SSFG_RS28220	SCN_RS03735, SCN_RS03725, SCN_RS03730, SCN_RS04530
	GTA	*TAC*	AAC, TAC	SCOt10	XNR_4542	SVEN_t11	SLI_10010	SSFG_RS24205	SCN_RS07625
	GTG	*CAC*	TAC, CAC	SCOt01, SCOt02	XNR_5317, XNR_5319	SVEN_t2, SVEN_t3	SLI_10003, SLI_10004	SSFG_RS28310, SSFG_RS28275,	SCN_RS03655, SCN_RS03665
Thr (T)	ACT	*AGT*	AGT, GGT	–	–	–	–	–	–
	ACA	*TGT*	AGT, TGT	SCOt47	XNR_2551	SVEN_t49	SLI_10051	SSFG_RS19050	SCN_RS15670
	ACC	*GGT*	AGT, GGT	SCOt49, SCOt61	XNR_1117, XNR_3695	SVEN_t53, SVEN_t64	SLI_10053, SLI_10065	SSFG_RS14320	SCN_RS17550, SCN_RS22525
	ACG	*CGT*	TGT, CGT	SCOt28	XNR_2697	SVEN_t30	SLI_10029	SSFG_RS15630	SCN_RS11750
Pro (P)	CCT	*AGG*	AGG, GGG	–	–	–	–	–	–
	CCC	*GGG*	AGG, GGG	SCOt63, SCOt64, SCOt65	XNR_0211, XNR_0244	SVEN_t67, SVEN_t68	SLI_10067, SLI_10068, SLI_10069	SSFG_RS03965, SSFG_RS04060, SSFG_RS503850	SCN_RS25645, SCN_RS26115
	CCA	*TGG*	AGG, TGG	SCOt16	XNR_4324	SVEN_t17	SLI_10016	SSFG_RS22915	SCN_RS08555
	CCG	*CGG*	TGG, CGG	SCOt29	XNR_2739	SVEN_t31	SLI_10030	SSFG_RS15825	SCN_RS12385
Ser (S)	TCT	*AGA*	AGA, GGA	–	–	–	–	–	–
	TCC	*GGA*	AGA, GGA	SCOt38	XNR_2859	SVEN_t40	SLI_10039	SSFG_RS18130	SCN_RS14915
	TCA	*TGA*	AGA, TGA	SCOt34	XNR_2906	SVEN_t36	SLI_10035	SSFG_RS17845	SCN_RS13655
	TCG	*CGA*	TGA, CGA	SCOt37	XNR_2882	SVEN_t39	SLI_10038	SSFG_RS18035	SCN_RS14685
Ser (Z)	AGT	*ACT*	ACT, GCT	–	–	–	–	–	–
	AGC	*GCT*	GCT	SCOt35	XNR_2899	SVEN_t37	SLI_10036	SSFG_RS17910	SCN_RS13580
Phe (F)	TTT	*AAA*	AAA, GAA	–	–	–	–	–	–
	TTC	*GAA*	GAA	SCOt42	XNR_2833	SVEN_t44	SLI_10043	SSFG_RS18285	SCN_RS15085
Met (M)	ATG	*CAT*	CAT	SCOt11, SCOt46, SCOt50, SCOt52, SCOt53	XNR_1595, XNR_1597, XNR_2506, XNR_2818, XNR_3696, XNR_4468	SVEN_t12, SVEN_t48, SVEN_t54, SVEN_t57	SLI_10011, SLI_10047, SLI_10054, SLI_10056, SLI_10057	SSFG_RS11885, SSFG_RS14315, SSFG_RS11875, SSFG_RS18340, SSFG_RS23665	SCN_RS07950, SCN_RS15180, SCN_RS17555, SCN_RS19765, SCN_RS19775
Tyr (Y)	TAT	*ATA*	ATA, GTA	–	–	–	–	–	–
	TAC	*GTA*	GTA	SCOt48	XNR_3691	SVEN_t52	SLI_10052	SSFG_RS14340	SCN_RS17530
His (H)	CAT	*ATG*	ATG, GTG	–	–	–	–	–	–
	CAC	*GTG*	GTG	SCOt19	XNR_4179	SVEN_t20	SLI_10019	SSFG_RS00915	SCN_RS09385
Gln (Q)	CAA	*TTG*	TTG	–	XNR_2059	SVEN_t27	–	SSFG_RS20490	SCN_RS10785,
	CAG	*CTG*	TTG, CTG	SCOt56, SCOt59	XNR_1260, XNR_1263	SVEN_t59, SVEN_t62	SLI_10059, SLI_10062	SSFG_RS10360, SSFG_RS10345	SCN_RS21830, SCN_RS21845
Asn (N)	AAT	*ATT*	ATT, GTT	–	–	–	–	–	–
	AAC	*GTT*	GTT	SCOt12, SCOt13	XNR_4469, XNR_4470	SVEN_t13, SVEN_t14	SLI_10012, SLI_10013	SSFG_RS23655, SSFG_RS23660	SCN_RS07955, SCN_RS07960
Lys (К)	AAA	*TTT*	TTT	SCOt45	XNR_2825	SVEN_t47	SLI_10046	–	SCN_RS15130
	AAG	*CTT*	TTT, CTT	SCOt20, SCOt21, SCOt22	XNR_1720, XNR_1739, XNR_1743	SVEN_t21, SVEN_t22, SVEN_t23	SLI_10020, SLI_10021, SLI_10023	SSFG_RS21980, SSFG_RS21890, SSFG_RS21910	SCN_RS09450, SCN_RS09510, SCN_RS09530
Asp (D)	GAT	*ATC*	ATC, GTC	–	–	–	–	–	–
	GAC	*GTC*	GTC	SCOt41, SCOt43	XNR_2832, XNR_2835	SVEN_t43, SVEN_t45	SLI_10042, SLI_10044	SSFG_RS18290, SSFG_RS18275	SCN_RS15055, SCN_RS15090
Glu (E)	GAA	*TTC*	TTC	SCOt44	XNR_2831	SVEN_t46	SLI_10045	SSFG_RS18295	SCN_RS15095
	GAG	*CTC*	TTC, CTC	SCOt57, SCOt58, SCOt60	XNR_1259, XNR_1261, XNR_1262	SVEN_t60, SVEN_t61, SVEN_t63	SLI_10060, SLI_10061, SLI_10063	SSFG_RS10340, SSFG_RS10355, SSFG_RS10350	SCN_RS21835, SCN_RS21840, SCN_RS21850
Cys (C)	TGT	*ACA*	ACA, GCA	–		–	–	–	–
	TGC	*GCA*	GCA	*cysT*	XNR_1833, XNR_5306	SVEN_t25, SVEN_t5	SLI_10006	SSFG_RS28225, SSFG_RS21415	SCN_RS03720, SCN_RS09880
Trp (W)	TGG	*CCA*	CCA	SCOt51	XNR_3704	SVEN_t55	SLI_10055	SSFG_RS14265	SCN_RS17590
Leu (L)	TTA	*TAA*	TAA	*bldA*	XNR_1995	SVEN_t26	SLI_10025	SSFG_RS20685	SCN_RS10610
	TTG	*CAA*	TAA, CAA	SCOt09	XNR_4869	SVEN_t10	SLI_10009	SSFG_RS25860	SCN_RS05770
	CTT	*AAG*	AAG, GAG	–	–	–	–	–	–
	CTC	*GAG*	AAG, GAG	SCOt08, SCOt62	XNR_0380, XNR_5150	SVEN_t1, SVEN_t66, SVEN_t9	SLI_10008, SLI_10066	SSFG_RS05545, SSFG_RS27585	SCN_RS04345, SCN_RS24980
	CTA	*TAG*	AAG, TAG	SCOt23	XNR_1776	SVEN_t24	SLI_10022	SSFG_RS21585	SCN_RS09675
	CTG	*CAG*	TAG, CAG	SCOt31	XNR_3043	SVEN_t33	SLI_10032	SSFG_RS17260	SCN_RS14270
Ile (I)	ATT	*AAT*	AAT, GAT	–	–	–	–	–	–
	ATC	*GAT*	AAT, GAT	SCOt33	XNR_3013	SVEN_t35	SLI_10034	SSFG_RS17370	SCN_RS14095
	ATA	*TAT*	AAT, GAT, TAT	–	–	–	–	–	–
Arg (R)	CGT	*ACG*	ACG, GCG	SCOt36	XNR_2898	SVEN_t38	SLI_10037	SSFG_RS17915	SCN_RS13575
	CGC	*GCG*	ACG, GCG	–	–	–	–	–	–
	CGA	*TCG*	ACG, TCG	–	–	–	–	–	–
	CGG	*CCG*	TCG, CCG	SCOt55	XNR_1483	SVEN_t58	SLI_10058	SSFG_RS11240	SCN_RS20605
	AGA	*TCT*	TCT	SCOt18	XNR_4258	SVEN_t19	SLI_10018	SSFG_RS22445	SCN_RS08865
	AGG	*CCT*	TCT, CCT	SCOt27	XNR_2176	SVEN_t29	SLI_10027	SSFG_RS20070	SCN_RS17025
Gly (G)	GGT	*ACC*	ACC, GCC	–	–	–	–	–	–
	GGC	*GCC*	ACC, GCC	SCOt39, SCOt40, *glyUa*	XNR_2848, XNR_2853, XNR_5307	SVEN_t4, SVEN_t41, SVEN_t42	SLI_10005, SLI_10040, SLI_10041	SSFG_RS18190, SSFG_RS18165, SSFG_RS28230	SCN_RS03715, SCN_RS14945, SCN_RS14970
	GGA	*TCC*	ACC, TCC	SCOt17	XNR_4323	SVEN_t18	SLI_10017	SSFG_RS02000, SSFG_RS18195, SSFG_RS34410, SSFG_RS22910	SCN_RS08560, SCN_RS14975
	GGG	*CCC*	TCC, CCC	SCOt30	XNR_3132	SVEN_t32	SLI_10031	*SSFG_RS06925, SSFG_RS16450*	SCN_RS12860

* Strains are abbreviated as follows: A3(2), *S. coelicolor* A3(2); J1074, *S. albus* J1074; ATCC 10712, *S. venezuelae* ATCC10712; ATCC14672, *S. ghanaensis* ATCC14672; ATCC 27064, *S. clavuligerus* ATCC27064

** Total number of tRNA genes in genome

**Fig. 1:**
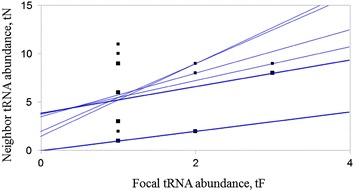
Correlation between a focal leucyl tRNA’s abundance tF and the abundance of its neighbors tN, across six *Streptomyces* genomes (see Additional file 1 for details). Each point represents a leucine tRNA species. The *solid lines* represent the regression lines between tF and tN for each genome. The data are dependent and nonrandom (Wilcox test, 0.042), and positively correlated (Spearman coefficient, min. 0.354). The mean of the distribution of correlation coefficient values for leucine codons differ from 0 (see Additional file 1: Fig. S2)

To gain initial insight into relative mistranslation rate associated with *bldA*, we applied a computational model of translation accuracy (Shah and Gilchrist 2010) that deduces ratio of abundances of cognate to near-cognate tRNAs (differ from cognate one by one mismatch; see Table 2). The rationale is that error rate would depend not only on the abundance of cognate tRNA, but also on the abundance of all near-cognates, that compete with the former for codon recognition. There was statistically significant positive correlation between the abundance of all leucyl tRNAs and their near-cognates for six *Streptomyces* species (Fig. 1), suggesting that error rates should not differ for different Leu codon-cognate tRNA pairs (if so, abundances of cognates and near-cognates would be uncorrelated). Similar correlation pattern was observed for most *Streptomyces* tRNAs (Additional file 1: Fig. S1, S2). We further calculated elongation and error rates for all six leucine codons and revealed that UUA had, in fact, the lowest missense error rate (Table 3). Our findings contrast general belief that low-abundant tRNAs are associated with higher mistranslation rates. Yet, they extend the nuanced view of codon accuracy, based originally on non-actinobacterial, low-GC (less than 70 %) genomes (Shah and Gilchrist 2010), onto GC-rich streptomycetes. Our data also agree with the expectation that proper operation of codon-based genetic switch should be based on accurate translation of UUA.

**Table 2:** Gene copy number (GCN) for tRNA genes in six *Streptomyces* genomes

	*S. coelicolor*		*S. albus*		*S. venezuelae*		*S. lividans*		*S. ghanaensis*		*S. clavuligerus*	
	tF	tN	tF	tN	tF	tN	tF	tN	tF	tN	tF	tN
*D*_*2*_												
F	1	11	1	12	1	13	1	9	1	12	1	13
Y	1	8	1	9	1	9	1	8	1	9	1	9
H	1	12	1	12	1	13	1	12	1	13	1	12
Q	2	10	3	14	3	14	2	10	3	16	2	15
N	2	12	2	12	2	12	2	12	2	12	2	12
K	4	17	4	19	4	17	4	17	4	18	4	18
D	2	16	2	16	2	17	2	14	2	16	2	14
E	4	15	4	16	4	16	4	15	4	16	4	17
C	1	8	2	8	2	8	1	8	2	8	1	8
*D*_*4*_												
A	4	29	4	28	5	28	4	27	5	32	4	30
V	6	28	6	29	6	29	4	28	6	33	4	29
T	4	27	4	27	4	26	4	27	4	27	4	26
P	5	19	4	20	4	22	5	19	5	20	5	20
G	5	22	5	23	5	24	5	20	5	24	5	24
*D*_*6*_												
S	4	27	4	28	4	28	4	27	4	28	4	27
L	6	25	6	26	7	24	6	23	7	26	6	26
R	4	19	4	20	4	18	4	19	4	22	4	20
*D*_*3*_												
I	1	16	1	17	1	16	1	14	1	15	1	17
*D*_*1*_												
M	5	10	6	10	4	10	5	10	4	10	5	10
W	1	6	1	7	1	7	1	6	1	8	1	7
*Leu*												
TAA	1	3	1	3	1	3	1	3	1	3	1	3
CAA	1	10	1	11	1	9	1	10	1	10	1	10
GAG	2	9	2	8	3	8	2	7	3	9	2	9
TAG	1	2	1	3	1	3	1	2	1	3	1	3
CAG	1	6	1	6	1	6	1	6	1	6	1	6

**Table 3:** Translation and mistranslation rates for six leucine codons in *Streptomyces*

Codon	Cognates	Pseudo-cognates	Near-cognates	*S. coelicolor*		*S. albus*		*S. ghananensis*		*S. clavuligerus*		*S. venezuelae*		*S. lividans*	
				Rc	Rn	Rc	Rn	Rc	Rn	Rc	Rn	Rc	Rn	Rc	Rn
CUA	UAG	AAG	CAG, GAG, UUG, UCG, UGG, UAA, UAU, UAC	2.067	0.007	2.067	0.009	2.067	0.009	2.067	0.009	2.067	0.010	2.067	0.007
CUC	GAG	AAG	UAG, CAG, GUG, GCG, GGG, GAA, GAU, GAC	4.134	0.014	4.134	0.014	4.134	0.012	4.134	0.014	6.201	0.012	4.134	0.011
CUG	CAG	UAC	AAG, UAG, GAG, CUG, CCG, CGG, CAA, CAU, CAC	2.068	0.019	2.068	0.020	2.068	0.019	2.068	0.019	2.068	0.019	2.068	0.019
CUU	AAG	GAG, UAG	CAG, AUG, ACG, AGG, AAA, AAU, AAC	–	–	–	–	–	–	–	–	–	–	–	–
UUA	UAA	AAA	CAA, GAA, UUA, UCA, UGA, UAU, UAC, UAG	2.067	0.006	2.067	0.006	2.067	0.006	2.067	0.006	2.067	0.006	2.067	0.006
UUG	CAA	UAA	AAA, GAA, CUA, CCA, CGA, CAU, CAC, CAG	2.068	0.014	2.068	0.015	2.068	0.014	2.068	0.014	2.068	0.013	2.068	0.014

Rather narrow options for UUA mistranslation, revealed by our analysis, did not take into account that decoding properties of tRNAs can be tuned via posttranscriptional modifications. We identified in genomes of two model streptomycetes a large set of genes for such modifications (including k^2^C; see above), seven of which are involved in maturation of nascent ${\text{tRNA}}^{\text{UAA}}{}_{\text{Leu}}$ in various non-actinomycete bacteria (Table 4 and Additional file 1: Fig. S3). Of particular interest are the genes for modification of anticodon loop and adjacent bases of ${\text{tRNA}}^{\text{UAA}}{}_{\text{Leu}}$ (see Additional file 1). For example, it is possible that a posttranscriptional modification of nascent *bldA* transcript important for UUA decoding and/or ${\text{tRNA}}^{\text{UAA}}{}_{\text{Leu}}$ maturation is delayed in *Streptomyces*. It would temporally limit the occurrence of translationally-competent ${\text{tRNA}}^{\text{UAA}}{}_{\text{Leu}}$, thus explaining late expression of TTA-containing genes. If so, then streptomycetes deficient in certain tRNA modification genes would resemble *bld* mutants. We are currently studying this idea using *S. albus* and *S. ghanaensis* as experimental models and invite verification of this conjecture for other strains. As a conclusion, our work shows that there are no theoretical grounds to consider UUA more error prone than the other leucine codons. We examined, *in silico*, options for UUA mistranslation and draw the attention of researchers to poorly understood aspects of function of *bldA* genetic switch.

**Table 4:** Genes for tRNA posttranscriptional modification in *S. coelicolor* and *S. albus* genomes

Protein	*S. coelicolor* A3(2) homolog	*S. albus* J1074 homolog	Annotation
IscS	SCO5486	XNR_1347	tRNAsulfurtransferase, PLP-dependent
IscU	SCO1920	XNR_4942	iron-sulfur cluster assembly enzyme
TruA	SCO4731	XNR_3758	tRNApseudouridine(38-40) synthase
TruB	SCO5709	XNR_1143	tRNApseudouridine synthase B
TruC	SCO1625	XNR_4806	tRNApseudouridine synthase C
DusB	SCO2497	XNR_4421	tRNA-dihydrouridine synthase B
TrmA	SCO5901	XNR_0992	tRNAm5(U34)methyltransferase
TrmB	SCO4111	XNR_2813	tRNA (guanine-N(7)-)-methyltransferase
TrmD	SCO5594	XNR_1214	tRNA m(1)G37 methyltransferase, SAM-dependent
TrmH	SCO4236	XNR_2558	tRNA mG18-2’-O-methyltransferase, SAM-dependent
RluA	SCO2073	XNR_4806	23S rRNApseudouridine(746), tRNApseudouridine(32) synthase, SAM-dependent
TadA	SCO4038	XNR_2881	tRNA-specific adenosine deaminase
FolE	SCO3403	XNR_3431	GTP cyclohydrolase I
QueA	SCO1804	XNR_5018	S-adenosylmethionine:tRNAribosyltransferase-isomerase
MnmA	SCO5488	XNR_1345	tRNA(Gln,Lys,Glu) U34 2-thiouridylase
MiaA	SCO5791	XNR_1074	delta(2)-isopentenylpyrophosphatetRNA-adenosine transferase
MiaB	SCO5787	XNR_1078	tRNA-i(6)A37 methylthiotransferase
AroA	SCO6819	XNR_1588	5-Enolpyruvylshikimate-3-phosphate synthetase
AroB	SCO1494	XNR_5357	3-Dehydroquinate synthase
AroC	SCO1496	XNR_5355	Chorismate synthase
AroE	SCO1498	XNR_5354	Dehydroshikimatereductase, NAD(P)-binding
AroD	SCO1961	XNR_4909	3-Dehydroquinate dehydratase
AroK	SCO1495	XNR_5356	Shikimate kinase I
TsaA	SCO5032	XNR_4120	tRNA-Thr(GGU) m(6)t(6)A37 methyltransferase, SAM-dependent
TsaB	SCO4750	XNR_3789	tRNA(ANN) t(6)A37 threonylcarbamoyladenosine modification protein; binding partner and protease for TsaD
TsaC	SCO5362	XNR_1471	t(6)A37 threonylcarbamoyladenosine biosynthesis protein
TsaD	SCO4752	XNR_3791	tRNA(ANN) t(6)A37 threonylcarbamoyladenosine modification protein; glycation binding protein
TsaE	SCO4747	XNR_3786	tRNA(ANN) t(6)A37 threonylcarbamoyladenosine modification protein; ADP binding protein
TilS	SCO3406	XNR_3428	tRNA(Ile)-lysidinesynthetase

## Additional file

10.1186/s40064-016-2683-6 Codon correlations and proposed Leu-tRNA modifications.
