# Supplementary material for: Decoding options and accuracy of translation of developmentally regulated UUA codon in Streptomyces: bioinformatic analysis
Source: Springerplus. 2016 Jul 4;5(1):982. doi: 10.1186/s40064-016-2683-6 (PMC4932002; doi:10.1186/s40064-016-2683-6)
Supplement: Supplementary file 1 — 10.1186/s40064-016-2683-6 Codon correlations and proposed Leu-tRNA modifications. [file 40064_2016_2683_MOESM1_ESM.pdf]

Electronic Supplementary Materials to the paper of Rokytskyy et al. “Decoding options and accuracy of translation of developmentally regulated UUA codon in *Streptomyces*: bioinformatic analysis”

Supplementary Methods

Identification of tRNA genes in genomes of Streptomyces coelicolor, S. albus, S. ghanaensis, S. clavuligerus, S. venezuelae and S. lividans *species*

Sequences of tRNA genes for genome of Streptomyces ceolicolor were taken from databases GtRNA-DB (Chan and Lowe 2009) and tRNADB-CE (Abe et al. 2014). Homologous sequences in genomes of *S. albus*, *S. venezuelae*, *S. lividans*, *S. ghanaensis* and *S. clavuligerus* were searched using NCBI Nucleotide database and *Streptomyces* genome server StrepDB. Further screening of the aforementioned genomes for tRNA genes has been carried out with online tRNA search tool tRNAscan-SE (Schattner et al. 2005). Potential isoacceptor tRNA were determined according to literature data (Lim and Curran 2001; Marck and Grosjean 2002; Grosjean et al. 2010). Prediction of tRNA decoding capacitites was based on work of dos Reis et al. (2004).

Mining Streptomyces coelicolor *and* S. albus *genomes for genes involved in tRNA modification*

Aminoacid sequences of enzymes for posttranscriptional tRNA modifications in bacteria were taken from MODOMICS database (Machnicka et al. 2013). Orthologs of these enzymes were identified in *Streptomyces* genomes using reciprocal best BLASTP hit strategy (Kuzniar et al. 2008).

Calculating the correlations between focal (cognate) and neighbor (near-cognate) tRNA abundances and mistranslation rates for Streptomyces

Procedure described in (Shah and Gilchrist 2010) has been followed. Equations 3 and 4 described by Shah and Gilchrist (2010) were used to calculate the rates of translation with cognate and near-cognate tRNAs. Certain parameters, such as specific protein synthesis rate and peptide chain elongation rate were taken from Shahab et al. (1996) and Cox (2004). We used fixed wobble penalties of w_RR/YY = 0.61 and w_RY/YR = 0.64. These parameters were taken from Curran JF, Yarus M (1989).

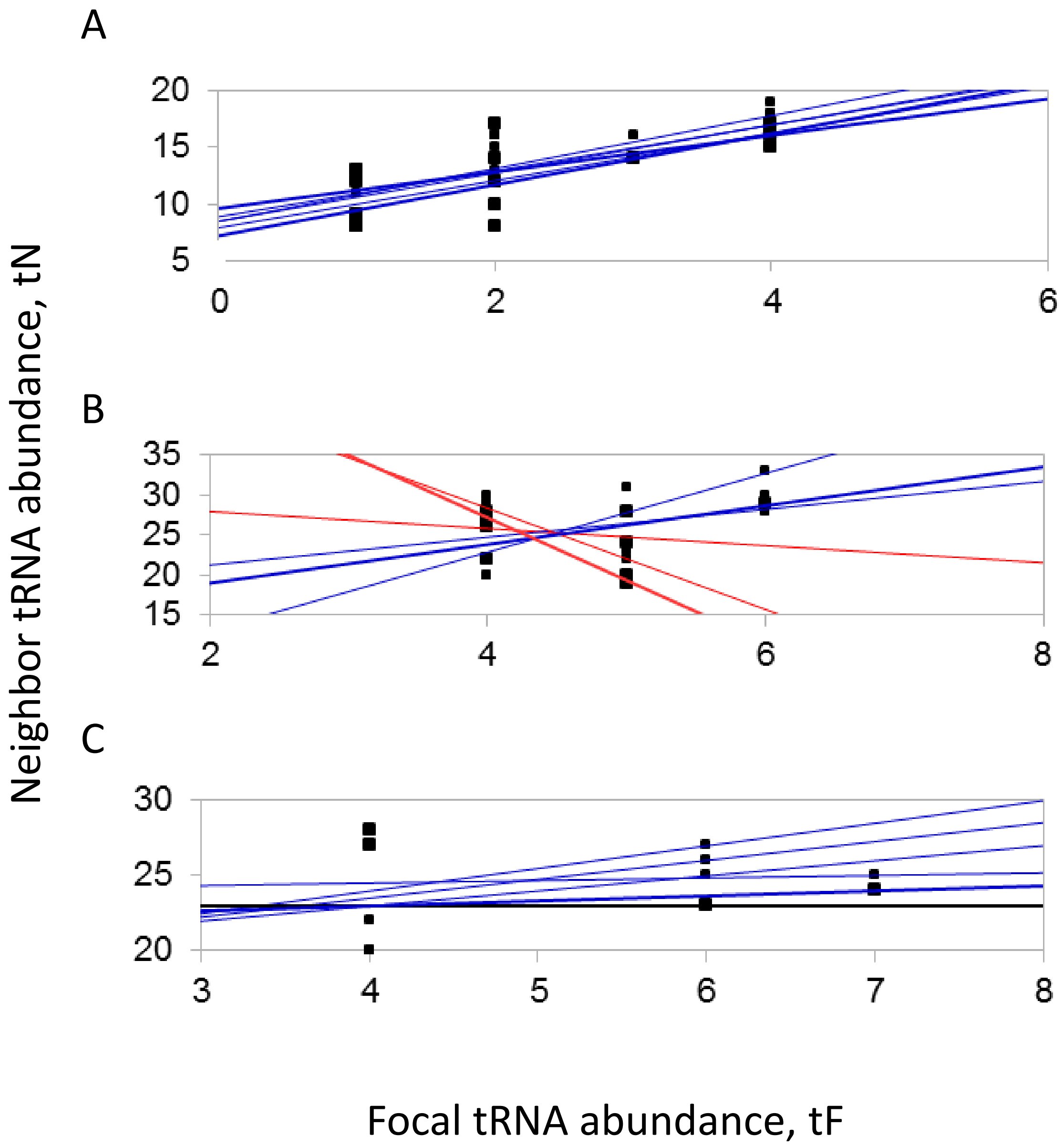

Fig. S1. Correlation between a focal tRNA's abundance tF and the abundance of its neighbors tN, across six *Streptomyces* genomes (see Table S1). Each point in panels represents a tRNA species that encodes an aminoacids with degeneracy (D_i) 2 (A), 4 (B) or 6 (C). The solid lines represent the regression lines between tF and tN for each genome. Blue lines represent positive correlation, red ones – negative correlation. The data are dependent and nonrandom (Wilcox test, 0.042), and weakly positively correlated (Spearman coefficient, min. 0.354) for set of codons from two-fold and six-fold degenerate groups (panels A and C).

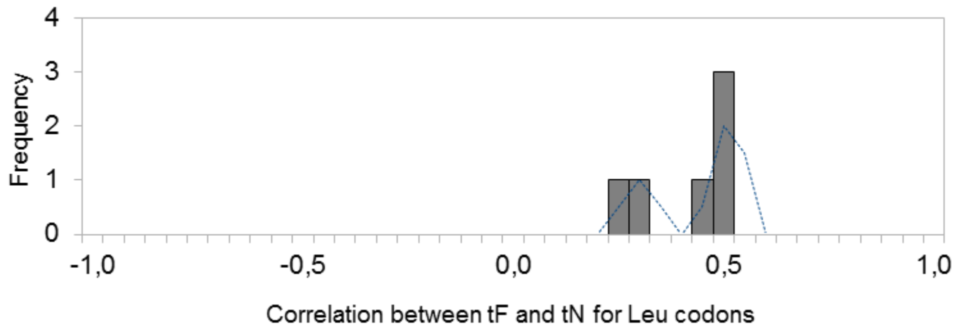

Fig. S2. The mean of the distribution of correlation coefficient values for leucine codons differ significantly from 0 (Wilcox test, $p < 0.05$).

A.

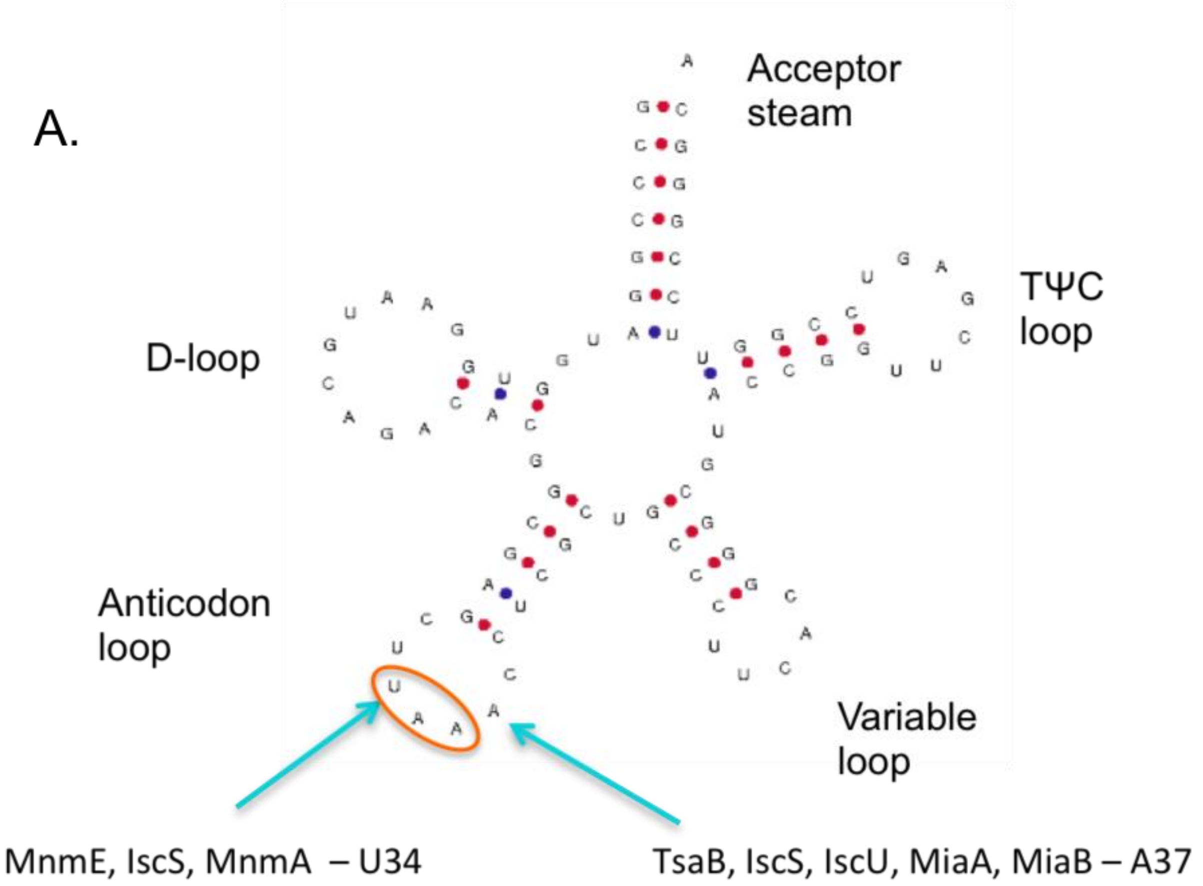

B.

```
bldA          GCCCGGATGGTGGAATGCAGACACGGCGAGCTTAAACCTCGCTGCCCCTTCGAGGGCGTG
XNR_1995      GCCCGGATGGTGGAATGCAGACACGGCGAGCTTAAACCTCGCTGCCCT-TCACGGGCGTA
SSFG_RS20685  GCCCGGATGGTGGAATGCAGACACGGCGAGCTTAAACCTCGCTGCCCCTGAGAGGGCGTG
              **********************************************    ...******.

bldA          CCGGTTCAAGTCCGGCTCCGGGCA
XNR_1995      CCGGTTCGAGTCCGGTTCCGGGCA
SSFG_RS20685  CCGGTTCAAGTCCGGCTCCGGGCA
              *******.******* ********
```

Fig. S3. A. Hypothetical secondary structures of tRNA$^{Leu}_{UAA}$ from *S. albus*. Anticodon position is circled. U34 and A37 positions of anticodon are marked with arrows. Enzymes known to be involed in postranscriptonal modification of these positions in model organisms (*E. coli*, *S. cerevisiae* etc) are listed at the bottom. **B.** Multiple sequence alignment of tRNA$^{Leu}_{UAA}$ genes from *S. coelicolor* (*bldA*), *S. albus* (XNR_1995) and *S. ghanaensis* (SSFG_RS20685). Anticodon position is circled

Supplementary References

Abe T, Inokuchi H, Yamada Y, Muto A, Iwasaki Y, Ikemura T (2014) tRNADB-CE: tRNA gene database well-timed in the era of big sequence data. Front Genet 5:114. doi: 10.3389/fgene.2014.00114.

Chan PP, Lowe TM (2009) GtRNAdb: a database of transfer RNA genes detected in genomic sequence. Nucleic Acids Res 37(Database issue):D93-7. doi: 10.1093/nar/gkn787.

Cox RA (2004) Quantitative relationships for specific growth rates and macromolecular compositions of *Mycobacterium tuberculosis*, *Streptomyces coelicolor* A3(2) and *Escherichia coli* B/r: an integrative theoretical approach. Microbiology 150(Pt 5): 1413 – 1426.

Curran JF, Yarus M (1989) Rates of aminoacyl-tRNA selection at 29 sense codons in vivo. Journal of Molecular Biology 209: 65–77.

dos Reis M, Savva R, Wernisch L (2004) Solving the riddle of codon usage preferences: a test for translational selection. Nucleic Acids Res. 32:5036-44.

Grosjean H, de Crécy-Lagard V, Marck C (2010) Deciphering synonymous codons in the three domains of life: co-evolution with specific tRNA modification enzymes. FEBS Lett 584(2):252-64. doi: 10.1016/j.febslet.2009.11.052.

Kuzniar A, van Ham RC, Pongor S, Leunissen JA (2008) The quest for orthologs: finding the corresponding gene across genomes. Trends Genet 24: 539–551.

Lim VI, Curran JF (2001) Analysis of codon:anticodon interactions within the ribosome provides new insights into codon reading and the genetic code structure. RNA 7: 942-57.

Machnicka MA, Milanowska K, Osman Oglou O, Purta E, Kurkowska M, Olchowik A, Januszewski W, Kalinowski S, Dunin-Horkawicz S, Rother KM, Helm M, Bujnicki JM, Grosjean H (2013) MODOMICS: a database of RNA modification pathways--2013 update. Nucleic Acids Res 41(Database issue):D262-7. doi: 10.1093/nar/gks1007.

Marck C, Grosjean H (2022) tRNomics: analysis of tRNA genes from 50 genomes of Eukarya, Archaea, and Bacteria reveals anticodon-sparing strategies anddomain-specific features. RNA. Oct;8(10):1189-232.

Sabi R, Tuller T (2014) Modelling the efficiency of codon-tRNA interactions based on codon usage bias. DNA Res 21(5):511-26. doi: 10.1093/dnares/dsu017.

Schattner P, Brooks AN, Lowe TM (2005) The tRNAscan-SE, snoscan and snoGPS web servers for the detection of tRNAs and snoRNAs. Nucleic Acids Res 1;33(Web Server issue):W686-9. doi: 10.1093/nar/gki366.

Shah P, Gilchrist MA (2010) Effect of correlated tRNA abundances on translation errors and evolution of codon usage bias. PLoS Genet 6:e1001128. doi: 10.1371/journal.pgen.1001128.

Shahab N, Flett F, Oliver SG, Butler PR (1996) Growth rate control of protein and nucleic acid content in Streptomyces coelicolor A3(2) and Escherichia coli B/r. Microbiology 142 (Pt 8):1927-35.

Stoletzki N, Eyre-Walker A (2007) Synonymous codon usage in *Escherichia coli*: selection for translational accuracy. Mol Biol Evol 24:374–381.
